# Outpatient Prescribing Patterns for Uncomplicated Cystitis in Premenopausal Women With and Without an Allergy to Guideline-Recommended Antimicrobials: A Retrospective Analysis

**DOI:** 10.7759/cureus.71000

**Published:** 2024-10-07

**Authors:** Darcy A Davis, Jaime A Foushee, Amber Stroupe, Shannon S Tiebout, Samantha Rikabi, Miles Lane

**Affiliations:** 1 Medical Student, Edward Via College of Osteopathic Medicine, Spartanburg, USA; 2 Pharmacology, Edward Via College of Osteopathic Medicine, Spartanburg, USA; 3 Internal Medicine-Pediatrics, Edward Via College of Osteopathic Medicine, Spartanburg, USA; 4 Family Medicine, Spartanburg Regional Healthcare System, Spartanburg, USA; 5 Statistics, Spartanburg Regional Healthcare System, Spartanburg, USA

**Keywords:** antimicrobial allergy, evidence-based medicine, guideline-recommended antimicrobials, uncomplicated cystitis, urinary tract infection

## Abstract

Objective: To determine if premenopausal women 18 to 50 years of age with uncomplicated cystitis who have an allergy to a guideline-recommended antimicrobial agent are less likely to receive guideline-preferred treatment compared to women without an allergy.

Methods: An electronic medical record report was used to identify females between the ages of 18-50 with a diagnostic code for acute uncomplicated cystitis at outpatient visits between December 1, 2017, through December 31, 2021. Patients with complicated urinary tract infections or pyelonephritis were excluded. After identifying the eligible encounters, a retrospective chart review was used to collect demographic information, known antimicrobial allergies, urinalysis and culture results, and prescribed antimicrobials. The primary outcome was a receipt of a guideline-preferred antimicrobial. Secondary outcomes included fluoroquinolone utilization and resistance patterns, as well as subgroup analyses of patients with specific allergies to guideline-preferred agents.

Results: A total of 496 patient encounters were screened for inclusion, with 165 meeting the inclusion criteria. Of those, 38 encounters were identified as having a documented antimicrobial allergy (23%) to a guideline-recommended agent. Demographic and clinical characteristics were similar between patients with and without antimicrobial allergies, except for race. Beta-lactam allergies were the most frequently documented antimicrobial allergy. All 165 patients received a guideline-recommended therapy, and 92 received a guideline-preferred agent (56%). Patient encounters without an antimicrobial allergy were numerically but not significantly more likely to receive guideline-preferred therapy (75 of 127 patients, 59%) compared to those with an allergy (17 of 38 patients, 45%; p=0.12). Patient encounters with an antimicrobial allergy were significantly more likely to receive a fluoroquinolone (FQ) prescription (n=6/38, 16%) compared to those without allergies (n=2/127, 1.6%; p=0.002). A subgroup analysis of patients with an allergy to a preferred agent was statistically less likely to receive a guideline-preferred treatment and more likely to receive an alternative agent. No statistical differences in other secondary outcomes were observed.

Conclusion: Premenopausal women with a documented antimicrobial allergy were statistically more likely to receive fluoroquinolone. Prescribers may be less likely to select a guideline-preferred treatment where antimicrobial allergies exist. Providers should be educated on evidence-based treatment within this population.

## Introduction

Urinary tract infections (UTIs) include a variety of bacterial infections impacting the urinary system. UTIs are differentiated based on the severity of infection and the anatomic location of the infection. Cystitis, an acute bacterial infection of the lower urinary tract affecting the urinary bladder and urethra, is one of the most common bacterial infections in women [1]. Approximately 11% of women annually and over 50% of women throughout their lifetime will be diagnosed with acute cystitis [2]. *Escherichia coli*, *Staphylococcus saprophyticus*, and other Enterobacteriaceae species, such as *Klebsiella* and *Proteus,* are the most common microbes associated with acute uncomplicated cystitis [3]. The gram-negative bacilli uropathogenic *E. coli *is responsible for an estimated 75% to 90% of episodes of acute uncomplicated cystitis and pyelonephritis [4,5].

The most current Infectious Diseases Society of America (IDSA) guidelines for the treatment of uncomplicated cystitis [6] recommend antimicrobial therapy to eradicate the infection, using an available agent not contraindicated due to allergy history. Preferred empiric treatment recommendations per these guidelines include nitrofurantoin, fosfomycin, trimethoprim-sulfamethoxazole (TMP/SMX), or pivmecillinam (unavailable in the United States until recently). The presence of antimicrobial allergies, altered renal function, availability and cost concerns, or community resistance patterns may preclude the use of one or more of these preferred treatment options. Beta-lactam antimicrobials, which have exhibited historically lower clinical efficacy in the literature, are an alternative option if preferred agents cannot be used. Fluoroquinolones (FQs) are additionally listed as an alternative treatment option and are generally considered a last-line therapy due to their propensity for “collateral damage” or severe adverse effects. Previous iterations of IDSA guidelines for treating uncomplicated cystitis have also advocated for reserving FQ use where recommended options are unable to be used due to historical costs associated with these agents and to delay the emergence of resistance from these broad-spectrum agents [7]. A growing prevalence of FQ-resistant urinary pathogens in adult patients presenting with UTI has been observed in the literature [8]. In more recent years, FQs have received additional boxed warnings and safety communications from the United States Food and Drug Administration, advising against the use of these agents in uncomplicated urinary tract infections and other less serious infections due to the risk associated with their use outweighing benefit where other treatment options are available [9]. Despite these warnings, FQ use for uncomplicated cystitis persists.

Across a variety of infection types, data suggest that patients with documented antimicrobial allergy labels are more likely to receive a broad-spectrum antimicrobial that often either has lower efficacy for the diagnosed infection or has an increased adverse effect profile, particularly well documented for penicillin and beta-lactam antibiotics [10,11]. While alternative first-line options exist, guideline concordance in patients with allergies to first-line antimicrobial agents in uncomplicated cystitis has not been examined. Patients with these allergies may be at increased risk of receiving a less preferred treatment and more likely to receive a FQ prescription. The objective of this study is to examine prescribing patterns of guideline-recommended antimicrobial agents for uncomplicated cystitis in pre-menopausal women in the context of documented allergies to these guideline-recommended therapies. The investigators hypothesize there will be guideline discordance more frequently observed in patients with allergies compared to those without. The primary outcome of the study is the receipt of guideline-recommended antimicrobials in patients with and without antimicrobial allergies. Secondary outcomes include examining FQ prescribing and resistance patterns within the source population, as well as subgroup analyses of patients with allergies to specific agents.

## Materials and methods

This study was a retrospective analysis of female subjects ages 18 to 50 years diagnosed with uncomplicated cystitis at the Center for Family Medicine outpatient clinics within the Spartanburg Regional Healthcare System between December 1, 2017, and December 31, 2021. The study was approved by the Spartanburg Regional Healthcare System Institutional Review Board (approval number 1711429-1). The aim of the study was to determine if premenopausal women between the ages of 18 and 50 years with an allergy to guideline-recommended antimicrobials are less likely to receive guideline-preferred treatments for uncomplicated cystitis compared to women without an allergy.

Patients were identified for screening via a report generated from the electronic medical record of women between the ages of 18-50 years with an outpatient diagnosis of one or more of the following International Classification of Diseases (ICD)-10 codes for UTI: ICD-10 CM: N30.0-acute cystitis without hematuria, N39.0-urinary tract infections, and R30.0-dysuria between the dates of 12/1/17 and 12/31/21. Inclusion criteria included a confirmed diagnosis by at least one documented urinalysis and receipt of antimicrobial therapy for the diagnosis. The presence of a positive nitrite test and/or leukocyte esterase test indicative of urinary tract infection was evaluated to confirm the diagnosis. In cases where a documented urinalysis was absent, inclusion was still possible if a clinical diagnosis of uncomplicated cystitis was made and antimicrobial therapy was initiated. Exclusion criteria for this study included patients with complicated UTIs, defined by documented structural genitourinary abnormalities, neurogenic bladder or self-catheterization, diabetes mellitus, or those with documented indwelling catheters. Additionally, individuals with pyelonephritis were excluded, identified by the presence of fever or systemic symptoms noted in the visit summary. 

Demographic information, including age, gender, race/ethnicity, clinical data, and lab values, was collected through an independent chart review of each included patient encounter. Clinical data collected included documented medication allergies and subsequent allergic reactions (if available), antimicrobial agent prescribed, urine culture with resulting pathogen grown and antimicrobial susceptibility profile, body mass index status, diabetic status, pregnancy status, and smoking status. The antimicrobial agent prescribed was categorized according to the drug class. A guideline-preferred treatment was based on the IDSA guidelines (Table 1) and considered if the patient was prescribed TMP/SMX, nitrofurantoin, fosfomycin, or pivmecillinam (not available in the United States during time of study). Antimicrobial susceptibility profiles were compiled where available. An isolate was considered not susceptible if the report indicated intermediate or resistant using Clinical and Laboratory Standards Institute (CLSI) breakpoints at the time of testing. Laboratory values collected included the glomerular filtration rate (GFR), blood urea nitrogen (BUN), and serum creatinine, which were obtained from the most recent metabolic panel within six months of the cystitis encounter, where available.

**Table 1:** Antimicrobial empiric treatment recommendations for acute uncomplicated cystitis [3] Standard dosing; may require individual dose adjustments. Reference no. [3]

Drug	Dosing*	Duration
Guideline preferred antimicrobials		
Nitrofurantoin monohydrate/macrocrystals	100 mg twice daily	5 days
Trimethoprim-sulfamethoxazole	160-800 mg twice daily	3 days
Fosfomycin	3 grams in one dose	1 day
Pivmecillinam	400 mg twice daily	5 days
Guideline alternative antimicrobials		
Fluoroquinolones		
Ciprofloxacin	250 mg twice daily	3 days
Levofloxacin	250 mg daily	
Beta-lactams		
Amoxicillin-clavulanate	500/125 mg twice daily	3-7 days
Cefdinir	300 mg twice daily	
Cefaclor	250-500 mg every 8 hours	
Cefpodoxime proexetil	100 mg twice daily	

Each encounter that met inclusion criteria was counted as a separate data entry; therefore, some patients were included in the data set more than once for subsequent infections. All data was de-identified following data collection for analysis. A priori-defined subgroup analyses were performed of subsets of patients with allergies specifically to guideline “preferred” antimicrobial agents and also for patients with documented sulfonamide allergies. Additionally, FQ utilization and resistance patterns were analyzed.

The primary outcome and secondary analyses were performed using Fisher’s Exact tests. Demographic data was analyzed using Chi-Squared or Fisher’s Exact tests for nominal data and using t-test, analysis of variance, or Wilcoxon rank-sum tests for continuous data. All analyses were performed with R developed with R Studio (www.rstudio.com). A p-value of <0.05 was considered statistically significant. 

## Results

**Figure 1:**
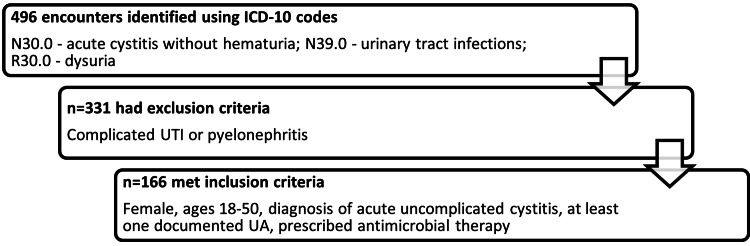
Consort diagram for the target population ICD: International Classification of Diseases, UTI: Urinary tract infection, UA: Urinalysis.

A total of 496 patient encounters were screened for inclusion, of which 165 met the inclusion criteria (Figure 1). An antimicrobial allergy was documented in 38 patient encounters (22.9%), the majority of which were listed to beta-lactams (n=22; 58%) followed by TMP/SMX (n=9; 24%), nitrofurantoin (n=5; 13%), and FQs (n=2; 5%). No patients had drug allergies to more than one drug class recommended in the treatment of uncomplicated cystitis.

Demographic information of the included study population is found in Table 2. Significant variation was present in the prevalence of antimicrobial allergy between age groups (p=0.002), with patient age groups 30-39 and 40-50 years having a higher prevalence of documented allergies to an antimicrobial compared to patients between the ages of 18-29 years. Additionally, there was significant variation in the prevalence of antimicrobial allergies based on documented race, with a higher prevalence of documented allergies occurring in patient encounters with a race documented as white or Caucasian (p=0.009). A total of 84 patients had laboratory values available for analysis, and while a statistically significant difference was observed in the most recent serum creatinine values between patient groups, all included patients with available lab values had a calculated glomerular filtration rate > 60 mL/min/1.73 m^2^.

**Table 2:** Demographic information ^1^Pearson’s Chi-squared test; Fisher’s exact test; Wilcoxon rank sum test. ^2^Pearson's Chi-squared test.​​​​​​​ ^3^Fisher's exact test. ^4^Wilcoxon rank sum test.

Variable	Antimicrobial Allergy n = 38	No Allergy n = 127	Overall N = 166	Test statistic^1^	p-value
Age Group (n=166)					
18-29	5 (13%)	56 (44%)	61 (37%)	12	0.002^2^
30-39	16 (42%)	32 (25%)	48 (29%)		
40-50	17 (45%)	39 (31%)	56 (34%)		
Race (n=65)					
Black	15 (39%)	62 (49%)	77 (47%)	9.4	0.009^2^
White	22 (58%)	43 (34%)	65 (39%)		
Other	1 (2.6%)	22 (17%)	23 (14%)		
Body mass index (n=165)					
>30.0 (Obese)	20 ( 53% )	63 ( 50% )	83 ( 50% )		0.56^3^
25.0-29.9 (Overweight)	11 ( 29% )	34 ( 27% )	45 ( 27% )		
18.5-24.9 (Normal)	6 ( 16% )	29 ( 23% )	35 ( 21% )		
<18.5 (Underweight)	1 ( 2.6% )	1 ( 0.8% )	2 ( 1.2% )		
Smoking status (n=165)					
Former smoker	10 (26%)	21 (17%)	31 (19%)	3.3	0.19^2^
Current smoker	9 (24%)	22 (17%)	31 (19%)		
Non-smoker	19 (50%)	84 (66%)	103 (62%)		
Markers of renal function (n=84)					
Serum creatinine	0.78 (0.76 – 0.86)	0.74 (0.65 – 0.78)	0.76 (0.67 – 0.81)	1,109	0.003^4^

A urine culture was obtained and available in the electronic medical record for 59% of included patient encounters (98/166). Eighty-four cultures yielded bacterial pathogen presence with 70 resulting in a specific isolate. *Escherichia coli* was the most commonly isolated pathogen on urine culture results for encounters included in the study (n=49/70; 70%), followed by *Klebsiella pneumoniae* (n=6; 8.6%), *Staphylococcus saprophyticus* (n=5; 7.1%), and *Proteus mirabilis* (n=4; 5.7%). Antimicrobial susceptibility testing was performed on 61 of the isolates, with the following resistance rates observed: 23% resistance to TMP/SMX (n=14/61), 6.6% to nitrofurantoin (n=4), and 8.2% to ciprofloxacin (n=5). An antimicrobial was prescribed for all included encounters. Overall, 100% of patient encounters resulted in prescribing any guideline-recommended antibiotic (n=165/165), where only 92 (56%) were prescribed a guideline-preferred antibiotic (TMP/SMX, nitrofurantoin, fosfomycin, pivmecillinam). Nitrofurantoin was the most commonly prescribed antimicrobial (n=60; 36%), followed by cephalexin (n=46; 28%), and TMP/SMX (n=32; 19%).

For the primary outcome, there was a numerical nonsignificant decrease in receipt of a guideline-preferred antimicrobial therapy in patients with antimicrobial allergies (17/38; 45%) compared to those without antimicrobial allergies (75/127; 59%), with a p-value of 0.12 (Pearson’s Chi-squared test).

Two of the secondary analyses were performed examining FQ utilization and resistance within the overall population (Table 3). Patients with an antimicrobial allergy were significantly more likely to receive an FQ prescription for treatment of uncomplicated cystitis compared to patients without allergies (p=0.002). A qualitative examination of the FQ prescriptions show six of the eight FQ prescriptions were for patients with an antimicrobial allergy to either a beta-lactam or TMP/SMX. In each case, an alternative guideline-recommended therapy was available for utilization. Of the 61 urine pathogens identified and tested for ciprofloxacin resistance, resistance was observed numerically more often in isolates obtained from patients with documented antimicrobial allergies to guideline- recommended agents (n=2/12;17%) compared to isolates obtained from patients without such allergies (n=3/49; 6.1%), although this finding was not statistically significant (p=0.25), likely due to a low number of isolates available for analysis within the study population.

**Table 3:** Secondary analyses: fluroquinolone-related outcomes ^1^Fisher's exact test. FQ: Fluoroquinolones.

	Antimicrobial Allergy	No Allergy	Overall	p-value
FQ prescribing (n=165)				
Prescribed FQ	6/38 (15.8%)	2/127 (1.6%)	8/165 (4.8%)	0.002^1^
Was not prescribed FQ	32/38 (84.2%)	125/127 (98.4%)	157/165 (95.2%)	
FQ resistance in isolated pathogens (n=61)				
Ciprofloxacin non-susceptible isolate	2/12 (17%)	3/49 (6.1%)	5/61 (8.2%)	0.25^1^
Ciprofloxacin susceptible isolate	10/12 (83%)	64/67 (94%)	56/61 (92%)	

Additionally, subgroup analyses were performed on patients with specific drug allergies. Receipt of a guideline-preferred therapy occurred statistically less often for patients with an allergy to any “preferred antimicrobial” (TMP/SMX, nitrofurantoin, fosfomycin, or pivmecillinam) n=4/14 (29%) compared to patients without such allergy (n=88/151; 58%, p=0.032).

## Discussion

The results of this study indicate a disparity in the prescribing of guideline-based antimicrobials for uncomplicated cystitis between premenopausal women with allergies to guideline-recommended antimicrobials compared to those without such allergies. While all patients in the study received a guideline-based treatment, patients with allergies were significantly more likely to receive an alternative treatment with a FQ. Additionally, patients with specific allergies to guideline-preferred treatments, notably TMP-SMX or nitrofurantoin, were significantly more likely to receive a non-preferred treatment regimen.

These findings are consistent with previous literature surrounding antimicrobial use and guideline adherence. Robinson and colleagues examined the appropriateness of empiric FQ use in comparison to nitrofurantoin use in five family medicine clinics and aimed to determine factors associated with FQ use [12]. Of 567 patients meeting inclusion criteria, 172 received an empiric FQ prescription and 395 received nitrofurantoin. Only 10.5% of FQ prescriptions were deemed appropriate, compared to 86.8% of nitrofurantoin prescriptions (p<0.01). The study identified four independent factors associated with FQ prescribing, including the prescribing clinic, patient age, prior urine culture within 12 months demonstrating resistance to nitrofurantoin, and recent nitrofurantoin use within 90 days prior to encounter. Notably, the clinic where the patient was treated emerged as the strongest predictor of antibiotic choice, emphasizing how individual clinic practice patterns may influence prescribing and highlighting a role for academic detailing within a clinical setting. While this study identified a continued disparity between guideline recommendations and antimicrobial prescribing, it did not examine the role antimicrobial allergies may play within this association.

Additional factors may play a role in prescriber adherence to guideline recommendations. Langner et al. [13] conducted a secondary analysis of data extracted from the National Disease and Therapeutic Index to examine guideline concordance in 44.9 million women treated for uncomplicated cystitis between 2015-2019, finding an overall concordance rate of 58.4% of treatments (95% CI 50.7-66). FQs were the most commonly prescribed agents (16.3 million; 36.4%), followed by nitrofurantoin and TMP/SMX, with FQ use declining over the observed study period. Guideline concordance was lower for women between the ages of 45-75 years and higher when the prescribing specialty was urology or obstetrics/gynecology. However, this study did not examine the role allergy to antimicrobial agents may play in guideline concordance.

While several factors identified in these studies suggest guideline discordance is commonly seen in the treatment of uncomplicated cystitis, our study findings add to the current body of literature, highlighting that despite where effective alternative guideline-based therapies are available, patients with allergies may be at increased risk for receipt of a non-guideline preferred therapy or a FQ. One potential explanation for this finding is that providers may become comfortable with prescribing a specific guideline-recommended therapy but are less familiar with alternative options when an allergy to that agent exists. Another potential source of guidance discordance may be a historical negative experience with a first-line medication in previous clinical experiences.

This study possessed several limitations that restrict generalizability. The study sample size was limited, with strict inclusion criteria. A total of 496 patient encounters were screened for inclusion criteria. Due to robust exclusion criteria as described in the study methodology, 165 patient encounters were eligible for study inclusion, with a small portion of patients having documented antimicrobial allergies. The exploration of the secondary analyses yielded very small sample sizes for analysis. However, these results are hypothesis generating for future research to further examine the trend towards increased FQ resistance observed on urinary culture in patients with antimicrobial allergies.

Another limitation to the generalizability of the study is the methodology of utilizing a retrospective chart review, collecting documented information from an electronic health record based on provider-documented ICD-10 codes and patient information. The study investigators were limited in collecting previously documented information that may have been inaccurate. Lastly, numerous factors are considered by a provider when selecting a specific antimicrobial agent, and patient factors outside of antimicrobial allergies that may have impacted a provider’s selection of an antimicrobial agent are not easily able to be identified from the study design, such as cost and availability concerns, antibiogram results, recent antibiotic use, or previous patient-specific urine culture susceptibilities.

As indicated by the study, patients with an antimicrobial allergy were less likely to receive guideline-preferred empiric antimicrobials for the treatment of acute uncomplicated cystitis despite alternative preferred treatment options being available and more likely to receive a FQ therapy. Several studies have examined strategies to enhance guideline-based prescribing, using a variety of strategies such as suppressing FQ susceptibilities on urine culture [14], multi-faceted educational training [15], and personalized prescribing feedback [16]. Focusing on strategies to enhance antimicrobial stewardship and provider education is warranted to improve rates of guideline-recommended antimicrobial prescribing, particularly in patients with antimicrobial allergies.

## Conclusions

Patients with an antimicrobial allergy were less likely to receive guideline preferred antimicrobial for the treatment of acute uncomplicated cystitis and significantly more likely to receive a FQ in an outpatient family medicine clinic setting. The results of this study indicate that guideline discordance may occur more frequently in patients with specific antimicrobial allergies, despite available alternative treatment options. Given the importance of evidence-based medicine and antimicrobial stewardship, future educational initiatives focused on optimal antimicrobial prescribing in this population may be warranted.
